# Advancements and Challenges in Bioelectrochemical Systems for Sustainable Nitrogen Removal from Wastewater

**DOI:** 10.3390/molecules31101745

**Published:** 2026-05-20

**Authors:** Songkai Qiu, Ruihao Li, Yu Zhang, Hanbing Zheng, Yuqun Zou, Zhuowei Cheng

**Affiliations:** 1 Zhejiang Key Laboratory of Low-Carbon Control Technology for Industrial Pollution, College of Environment, Zhejiang University of Technology, Hangzhou 310014, China; 2 Haina-Water Engineering Research Center, Yangtze Delta Region Institute of Tsinghua University, Jiaxing 314006, China

**Keywords:** bioelectrochemical systems, nitrogen removal, wastewater, challenges

## Abstract

Bioelectrochemical systems (BESs) for nitrogen removal, which integrate electrochemical processes with biological nitrogen removal technologies, is a sustainable and energy-efficient alternative to conventional biological nitrogen removal methods. However, the practical application of BESs for nitrogen removal remains limited due to technical challenges, and existing reviews have not provided an integrated analysis that systematically links mechanistic understanding, operational optimization, and engineering-scale challenges across multiple BES configurations. Therefore, the mechanisms and performance of main nitrogen removal pathways in BESs are critically summarized and compared within a unified framework. Low nitrogen removal rate and long-term instability are identified as the main challenges. Furthermore, the links between nitrogen removing performance and the key operational parameters, including substrate concentration, applied voltage, electrode materials and microbial interactions are systematically analyzed to reveal their influences and optimization opportunities. Finally, based on this integrated analysis, alternatives are proposed to improve the nitrogen removal rates and stability of BESs for nitrogen removal.

## 1. Introduction

Nitrogen removal is a critical process in wastewater treatment, primarily addressing the environmental risks of eutrophication and greenhouse gas emissions caused by excess nitrogen compounds [1]. Conventional biological methods, such as nitrification–heterotrophic denitrification and anaerobic ammonium oxidation (Anammox), face inherent challenges including slow microbial growth (e.g., Anammox doubling time of 11 days) [2], high energy consumption, and dependence on organic carbon sources for heterotrophic denitrification [3]. The biological yield coefficient of sugar-rich carbon sources is high, which will increase sludge production and disposal costs [4]. These limitations have driven the exploration of innovative technologies to enhance efficiency and sustainability.

The latest advancements in bioelectrochemical systems (BESs) have opened new pathways for nitrogen removal by integrating microbial metabolism within electrochemical systems. BESs can efficiently treat low-organic-content wastewater while reusing existing structures, significantly reducing infrastructure investment [5]. A BES utilizes extracellular electron transfer (EET) and electrode-driven redox processes to overcome traditional bottlenecks. Intermittent charge stimulation with the applied voltages of 0.6–1.0 V has been shown to enhance Anammox activity by altering membrane morphology and upregulating key enzymes (e.g., hydrazine synthase), achieving total nitrogen (TN) removal rates up to 6468 g N/m^3^/d [6]. Electrode-enhanced sequencing batch reactors (ESBR) reduced Anammox start-up time by 10 days compared to conventional systems [7]. Studies also demonstrate the feasibility of electro-stimulation enhanced ammonium (NH_4_^+^) removal in ferric ammonium oxidation (Feammox) process, achieving a removal rate of 80.62 g N/m^3^/d and 800 times less energy consumption than conventional process [8].

Despite these advancements, most BES-based nitrogen removal studies remain at the lab-scale stage using synthetic wastewater rather than real wastewater, and practical engineering applications are still limited [9]. The primary reasons for this are the diversity of BES configurations for nitrogen removal and the varying effectiveness when treating actual wastewater. Therefore, process selection must be tailored to specific water quality conditions and treatment objectives [10]. Furthermore, the factors affecting the nitrogen removal efficiency and long-term operational stability of such systems are complex, including electrode-related factors (e.g., electrode materials, applied voltage) [11,12], operational conditions (e.g., power supply mode) [13], substrate and water quality characteristics (e.g., pH, carbon source, nitrogen forms and concentrations) [14], and microbial factors. Systematic understanding of the key influencing parameters and optimal operating conditions for different BES configurations is still limited, which leaves the scalability and adaptability of BES technologies for nitrogen removal to real-world conditions underexplored [15,16].

Several recent reviews have substantially advanced our understanding of BESs for nitrogen removal, but each focuses on only a specific aspect of the topic. Khanthong et al. reviewed BESs for nitrogen removal from the perspective of carbon neutrality and H_2_ recovery, identifying bioelectrochemical–Anammox as the most promising pathway. However, this review did not systematically compare multiple BES pathways for nitrogen removal within a unified operational framework [17]. Liu et al. specifically addressed BES-enhanced nitrogen removal from carbon-deficient wastewater, focusing on combined denitrification pathways, electrode material modification, and N_2_O emission control. While valuable in these scopes, this review paid limited attention to nitrogen removal pathways such as extracellular electron transfer driven Anammox [18]. Zhu et al. provided a detailed overview of basic nitrogen removal principles in BESs and introduced the possible pathways of anodic and cathodic denitrification. However, while comprehensive in fundamental mechanisms, this review did not provide cross-configuration performance comparisons or systematic operational optimization across different nitrogen removal pathways [19]. Ponce-Jahen provided a detailed biochemical and metabolic description of the Anammox process with alternative electron acceptors, including iron, carbon materials, and BES electrodes (anodic Anammox). However, this review was exclusively focused on anammox-based BESs without integrating other nitrogen removal configurations [20]. Paul and Banerjee presented a broad introductory overview of both conventional biological nitrogen removal processes and BESs, but did not provide a critical analysis linking mechanistic understanding with operational optimization and engineering challenges [21].

Despite these significant contributions, existing reviews predominantly concentrate on either fundamental mechanisms, specific process configurations, or laboratory scale performance, rather than providing an integrated analysis that systematically links mechanistic understanding, operational optimization, and practical engineering challenges. Therefore, the present review focuses on: (1) the mechanistic integration and performance analysis of five representative BES configurations for nitrogen removal, including Anammox-based BES, Feammox-based BES, nitrifying BES, cathodic denitrifying BES, and combined processes, with emphasis on the unique bottleneck of each configuration; (2) the systematic evaluation of key operational parameters including substrate concentration, applied voltage, electrode material selection, and microbial interactions, with cross-configuration comparisons; and (3) the critical analysis of engineering-scale challenges. Based on this analysis, actionable alternatives are proposed to enhance nitrogen removal rates and operational stability, thereby providing a roadmap for scaling BES toward sustainable and practical wastewater treatment.

## 2. Bioelectrochemical Nitrogen Removal Processes

Five key BES configurations for nitrogen removal are summarized in Table 1, including (1) EET-dependent Anammox systems that directly utilize electrodes to mediate electron transfer, enabling NH_4_^+^ removal without NO_2_^−^ involvement; (2) Feammox-based BES enabling the oxidation of NH_4_^+^ with the reduction of Fe(III); (3) nitrifying BES, in which nitrifying bacteria utilize electrodes as electron acceptors, driving efficient ammonia oxidation; (4) cathodic denitrifying BES employ electrons from the cathode or electrolytically produced hydrogen to achieve denitrification with low carbon dependency, and (5) combined processes integrate the above multiple pathways to establish synergistic nitrogen removal networks. BESs that use electrical charge to enhance microbial activity and electrolyze water to provide oxygen for nitrifying bacteria are also discussed. The five BES configurations described below are intended as representative groupings to organize the discussion of distinct nitrogen removal pathways and mechanisms. These categories are not strictly mutually exclusive, as practical systems may combine features from multiple configurations within a single reactor. Therefore, some overlap among categories is expected in engineered systems.

### 2.1. Anammox-Based BES

**Figure 1:**
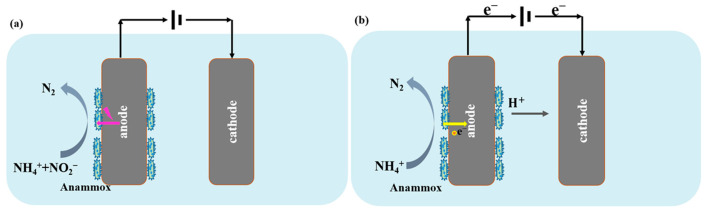
Scheme of Anammox-based BES. (**a**) Electricity stimulated pathway of Anammox-based BES. (**b**) Extracellular electron transfer (EET)-dependent Anammox.

Traditional Anammox process uses NO_2_^−^ as an electron acceptor to oxidize NH_4_^+^. However, the enrichment of Anammox bacteria remains challenging due to their slow growth rate, with a doubling time of approximately 11 days [2]. Furthermore, a substantial amount of energy is used for the initial oxidation of NH_4_^+^-N, which constrains their theoretical maximum growth yield to a low range of 0.13–0.16 g biomass/g NH_4_^+^-N [25]. Electrical stimulation has been reported to be effective in reducing the time for the startup of Anammox process in a BES (Figure 1). Studies have demonstrated that applied voltage enabled the rapid startup of Anammox process. In an electrode-ESBR with a current density of 0.10 mA cm^−2^, the startup of Anammox process was achieved within 91 days, representing a 10-day reduction compared with conventional sequencing batch reactors (SBR) without current application [7]. Sun et al. [28] revealed that the charging mode changes Anammox bacteria’s metabolic pathway and duration of startup: stepwise increased voltage promoted NH_4_^+^ oxidation to dinitrogen gas (N_2_) via hydrazine (N_2_H_4_) as the intermediate, whereas constant voltage operation used hydroxylamine (NH_2_OH) as the intermediate. This stepwise increased voltage enabled rapid start-up and effective treatment of low NH_4_^+^-N wastewater, achieving NH_4_^+^-N removal rates up to 84%.

Applying an external voltage also enhances the nitrogen removal performance of Anammox-based BES by influencing the permeability of the Anammox cell membrane, and simultaneously increasing the enzyme activity (Table 2). For instance, exposure to a low electrostatic field (0.6–1.0 V) alters the membrane morphology of Anammox cells, thereby improving mass transfer and upregulating key enzyme activities (e.g., hydrazine synthase). Under a 6 h application-rest cycle, the TN removal rate reached approximately 6468 g N/m^3^/d, demonstrating the efficacy of intermittent electrochemical stimulation [6].

Nevertheless, the above-mentioned electricity stimulated pathway of the Anammox-based BES still relies on stable NO_2_^−^ supply, which is one of the main challenges for an Anammox-based process. Emerging evidence proves that Anammox possesses multi-heme cytochromes and can carry out the EET process [42]. This means in BESs, Anammox bacteria attaching to the anode can oxidize NH_4_^+^ to generate electrons, and transfer these electrons to the anode and finally to the cathode through an external circuit (Figure 1). This EET-dependent pathway converts NH_4_^+^ to N_2_ via NH_2_OH intermediates without using NO_2_^−^ and without producing NO_3_^−^ [1]. Kadam et al. systematically evaluated NH_4_^+^ removal in a single-chamber BES across varying NH_4_^+^:NO_2_^−^ ratios (1:1, 1:0.5, 1:0). At 0.4 V vs. Ag/AgCl, 57.86% of NH_4_^+^ removal was observed in reactor R-3 without NO_2_^−^ supply [31]. Using a modified carbon brush as anode was reported to enhance nitrogen removal for Anammox-based BESs, where at the voltage of 0.8 V, a maximum NH_4_^+^-N removal of 41% with a coulombic efficiency of 40.9% were achieved without the addition of NO_2_^−^ [38]. Comparative analysis by Wang et al. revealed that while EET-dependent reactors achieved 93.2% nitrogen removal efficiency, their nitrogen loading capacity was not comparable with conventional nitrite-dependent systems [30].

**Figure 2:**
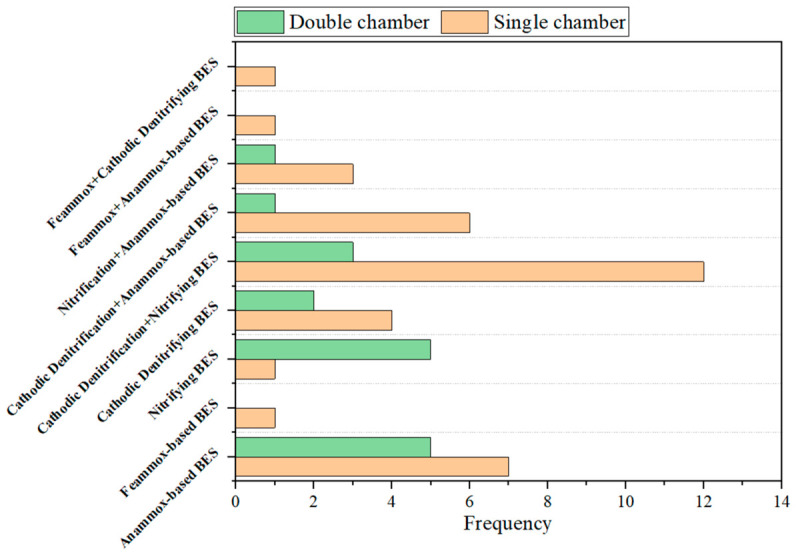
Frequency of single chamber and double chamber configurations in BES for nitrogen removal. Please see Section 3 for the detailed dataset selection criteria.

Overall, two mechanistically distinct approaches were used within Anammox-based BESs: (1) electricity-stimulated conventional Anammox, in which applied voltage accelerates startup and enhances enzymatic activity while still relying on NO_2_^−^ supply; and (2) EET-dependent Anammox, which bypasses the requirement for NO_2_^−^ entirely by using the anode as the terminal electron acceptor. The first approach offers more immediate operational benefit by reducing startup time and improving TN removal rates under optimized voltage conditions. By contrast, the second approach is conceptually more innovative but currently constrained by far lower nitrogen removal rates (12–56 g N m^−3^ d^−1^) compared to both conventional heterotrophic denitrification (80–1360 g N m^−3^ d^−1^) and traditional Anammox (up to 76.7 kg N m^−3^ d^−1^) [43]. However, neither approach has demonstrated the long-term operational stability required for engineering applications, with many studies operated for only days to weeks and exhibiting considerable performance fluctuation as reflected in Table 2. Compared to a Feammox-based BES, nitrifying BES, and cathodic denitrifying BES, a single-chamber Anammox-based BES has received the most research attention (Figure 2); yet, this has not translated into resolved challenges regarding removal rate and stability.

### 2.2. Feammox-Based BES

The conventional Feammox process is an autotrophic nitrogen removal pathway that occurs under strictly anaerobic conditions. In this process, Fe(III) compounds such as Fe_2_O_3_ serve as electron acceptors, and iron-reducing bacteria oxidize NH_4_^+^ to N_2_, NO_2_^−^, or NO_3_^−^, while generating Fe(II). Although this process can achieve anaerobic NH_4_^+^ removal with a maximum reported rate of up to 62.28 g N m^−3^ d^−1^, the presence of organic carbon as a competing electron donor can inhibit NH_4_^+^ removal efficiency. Moreover, the reliance on external Fe(III) addition and the slow re-oxidation of Fe(II) result in inefficient iron cycling, which limits long-term operational stability and hinders further improvement in nitrogen removal performance [22].

Qing et al. noted that the NH_4_^+^ conversion efficiency in the Feammox process is largely limited by the availability of Fe(III). Therefore, developing effective strategies to enhance Fe(II)/Fe(III) cycling and reuse has recently become a key research focus in Feammox studies [44]. Studies have shown that applying a constant voltage of 0.6 V in a BES enabled the continuous supply of Fe(III) for Feammox, which was achieved by the valence cycling of solid iron driven by anode potential. The Fe(III) content increased from an initial level of approximately 0 mM to 99 ± 22 mM, while the Fe(II) content decreased correspondingly, thereby continuously supplying electron acceptors for autotrophic nitrogen removal by Feammox bacteria [45]. The electrochemically enhanced Feammox process employing low applied voltage and Fe_2_O_3_ addition has been reported to enrich electroactive functional microbial consortia, thereby improving electron transfer and substrate oxidation efficiency while promoting efficient Fe(II)/Fe(III) cycling. This reduces dependence on external iron and weakens carbon-source competition due to electrochemically driven electron transfer. In an anaerobic single-chamber reactor operated at a constant voltage of 0.5 V with Fe_2_O_3_ supplementation, the system enriched electroactive functional bacteria (e.g., *Geobacter*, *Pseudomonas*, and *Nitrosomonas*) under low organic carbon conditions (COD 50 mg/L). This enrichment enhanced Fe(II)/Fe(III) cycling and electron transfer, leading to efficient ammonia-nitrogen (NH_4_^+^-N) removal at a rate of 80.62 g N m^−3^ d^−1^ with a specific energy consumption of only 0.002 kWh kg^−1^ N [8]. In addition to the Feammox process discussed above, Fe(II)-driven autotrophic denitrification is a pathway of increasing relevance to BESs. This process can be directly integrated with BES configurations: the produced Fe(III) (hydr)oxides can be electrochemically reduced back to Fe(II) at the cathode, thereby closing the iron cycle and enabling sustained autotrophic denitrification with minimal external Fe(II) addition [46,47,48].

In summary, a Feammox-based BES resolves the primary bottleneck of the conventional Feammox process, the limited and unsustainable supply of electron acceptors, by producing Fe(III) from Fe(II) via anode-driven valence cycling. This represents a functional distinction from other BES nitrogen removal pathways that rely on the microbial electron transfer to electrode. The reported energy consumption of 0.002 kWh kg^−1^ N is notably lower than conventional electrochemical nitrogen removal processes, which is encouraging for energy sustainability. However, the number of published studies on Feammox-based BESs remains limited compared with the other configurations (Figure 2). Furthermore, the sensitivity of the system to organic carbon competition also introduces significant uncertainty about its performance robustness under real wastewater conditions. Further investigation of microbial community stability and Fe cycling efficiency under variable influent compositions and operating conditions such as hydraulic retention times is required for the upscaling of this technology [46].

### 2.3. Nitrifying BES

**Figure 3:**
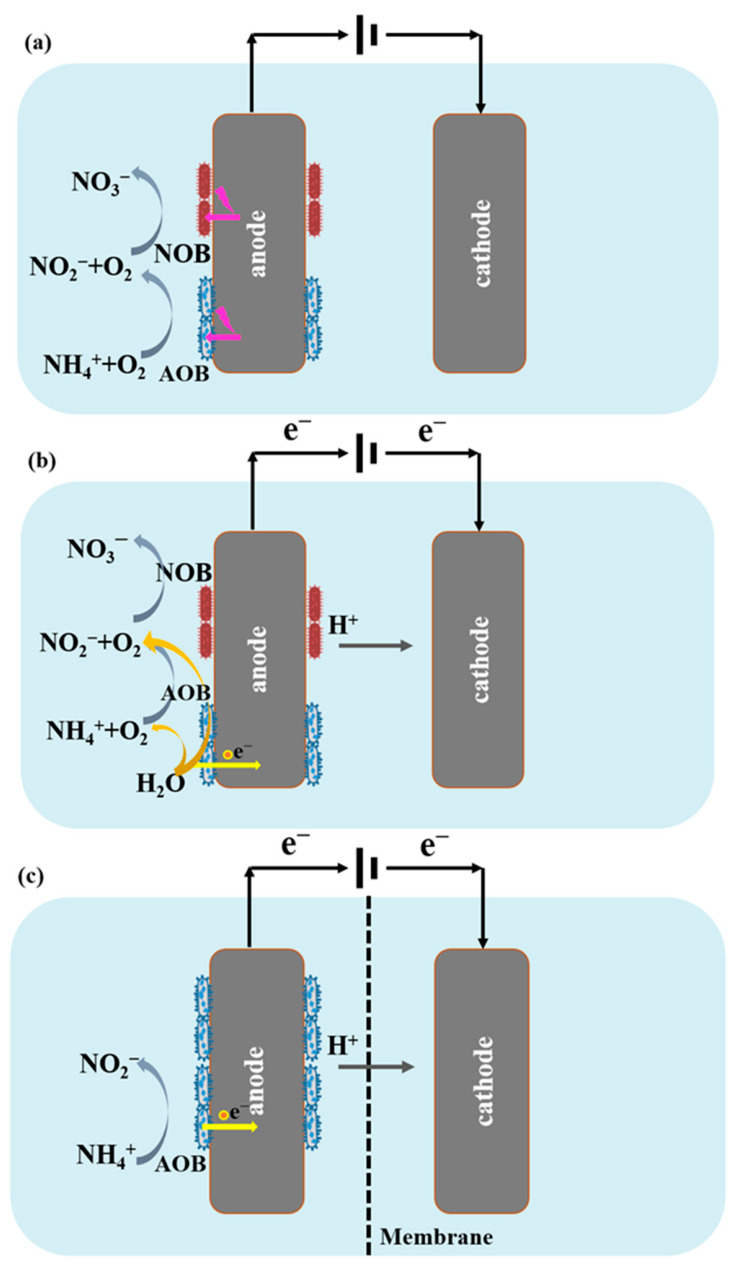
Scheme of nitrifying BES. (**a**) Enhanced nitrification with electricity stimulation. (**b**) Nitrification coupling water electrolysis. (**c**) Extracellular electron transfer (EET)-dependent Nitrification. AOB: ammonia-oxidizing bacteria. NOB: nitrite-oxidizing bacteria. Pink arrows: electrical stimulation pathways. Red arrows: extracellular electron transfer dependent pathways; Green arrows: pathways based on electrolysis of water.

In traditional NH_4_^+^-N treatment processes, nitrification serves as the initial stage, by aerobically oxidizing NH_4_^+^-N to NO_2_^−^ and subsequently NO_3_^−^ [3,49]. The performance of traditional nitrification process can be improved by coupling with BESs. Three nitrification processes implemented in BESs are summarized and illustrated in Figure 3. The first pathway is to enhance performance using external electrical stimulation (Figure 3a). In a single-chamber BES, applying a low voltage of 0.2–0.4 V to an anode attached with nitrifying biofilm enhanced electron transfer from NH_4_^+^ to NO_2_^−^, thereby significantly increasing NH_4_^+^-N removal efficiency from 70.3% to 92.6% as the applied voltage was raised [15]. Additionally, with advances in bioelectrochemical technology, BESs based on water electrolysis have been explored for biological nitrogen removal, as is shown in Figure 3b [50]. BESs can generate oxygen through water electrolysis and thus create a suitable microenvironment for nitrifiers. Consequently, nitrifying bacteria attached to the anode surface utilize the oxygen produced in situ via water electrolysis to achieve NH_4_^+^ oxidation [51].

However, water electrolysis requires a theoretical minimum applied voltage of 1.23 V under standard conditions. In practical systems, an even higher voltage is needed due to overpotentials and ohmic losses, which leads to increased energy consumption. In BESs, the oxidation of NH_4_^+^-N by nitrifying bacteria can be realized without the presence of molecular oxygen. A dual-chamber BES using the anode as an electron acceptor realized anode-driven NH_4_^+^ oxidation based on nitrifying bacteria *Nitrosomonas* europaea [23]. In this study, NO_3_^−^ was the main product, accounting for approximately 95% of the NH_4_^+^ consumed, while a small amount of NO_2_^−^ was also detected. Furthermore, the anaerobic oxidation of NH_4_^+^-N to N_2_ by nitrifying bacteria and other denitrifying bacteria in nitrifying BESs was also reported. Vilajeliu-Pons et al. [24] demonstrated that NH_4_^+^ under anerobic conditions could be completely converted to N_2_ with minimal accumulation of NO_2_^−^/NO_3_^−^/N_2_O in a continuous-flow BES. The removal rate reached 35 ± 10 g-N m^−3^ d^−1^, while energy consumption was reduced 35-fold. In this approach, the anode substitutes oxygen as the electron acceptor. *Nitrosomonas* electrochemically oxidizes NH_4_^+^ to NH_2_OH, which is subsequently further converted to N_2_ within the anode biofilm through the synergistic action of a complex microbial community.

Taken together, the three nitrification pathways implemented in BESs represent a progression from incremental enhancement to fundamental mechanistic innovation. Electrical stimulation is the most immediately practicable approach, offering modest but consistent improvements in NH_4_^+^-N removal efficiency at low applied voltages (0.2–0.4 V). Water electrolysis-based oxygen supply offers operational independence from external aeration but requires higher applied voltages. The EET-dependent pathway is the most transformative, enabling complete NH_4_^+^ conversion to N_2_ without dissolved oxygen and with substantially reduced energy consumption. However, it depends on a complex, precisely structured microbial community and remains poorly understood in terms of the specific electron transfer mechanisms involved. Compared to other configurations such as an Anammox-based BES, a nitrifying BES produces NO_2_^−^ or NO_3_^−^ rather than N_2_ unless coupled with downstream denitrification. This necessitates subsequent treatment steps unless the EET-dependent pathway is applied.

### 2.4. Cathodic Denitrifying BES

Heterotrophic denitrification is a primary biological denitrifying pathway, in which heterotrophic denitrifying bacteria (HDB) reduce NO_3_^−^ and NO_2_^−^ to gaseous nitrogen using organic carbon sources (e.g., methanol and acetate) as electron donors [3,52]. Under anoxic conditions, the carbon-to-nitrogen (C/N) ratio required to sustain heterotrophic denitrification typically ranges between 3 and 5 [53]. However, for wastewater with low C/N ratio, external organic carbon supplementation is necessary for this traditional method. This practice not only substantially increases operational costs but also risks secondary pollution due to residual organic compounds.

**Figure 4:**
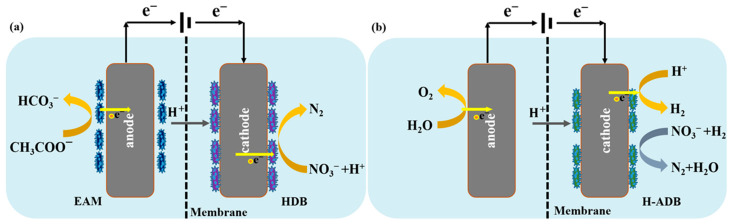
Scheme of cathodic denitrification processes. (**a**) Heterotrophic denitrification via extracellular electron transfer (EET). (**b**) Hydrogenotrophic denitrification powered by water electrolysis. EAM: electroactive microorganisms. HDB: heterotrophic denitrifying bacteria. H-ADB: hydrogenotrophic-autotrophic denitrifying bacteria.

Cathodic autotrophic denitrification has emerged as a sustainable alternative to address these limitations [27], in which the cathode can be employed in an electrochemical system as a solid electron donor for the reduction of NO_3_^−^ to N_2_ [54]. Studies have demonstrated that BESs enabled direct electron transfer from the cathode to denitrifying biofilms. Denitrifying bacteria, e.g., heterotrophic denitrifiers and hydrogenotrophic-autotrophic denitrifying bacteria (H-ADB), attached to the cathode can directly employ electrons transferred through the external circuit as electron donors for denitrification [55]. Figure 4 illustrates the heterotrophic denitrification and hydrogen based autotrophic denitrification processes within BESs. For instance, Clauwaert et al. employed a dual-chamber microbial fuel cell (MFC) in which the cathode heterotrophic denitrifying biofilm utilized electrons generated from acetate oxidation by anode microorganisms to directly reduce NO_3_^−^ completely to N_2_, without requiring external electrical energy or hydrogen as an intermediate. A maximum denitrification rate of 0.146 kg NO_3_^−^-N m^−3^ d^−1^ was achieved at the voltage of 0.075 V [26]. Compared to organic electron donors, molecular hydrogen (H_2_) serves as a cost-effective electron donor [56]. BESs can effectively enhance hydrogen autotrophic denitrification under low-carbon conditions [57]. In the cathode of a BES, hydrogenotrophic denitrifiers remove NO_3_^−^ using H_2_ generated from water electrolysis or through H^+^ reduction [58]. Zhu et al. used a three-dimensional biofilm electrode. Under a current of 40 mA, the rate of H_2_ production by water electrolysis optimally matched the metabolic demand of H-ADB, providing sufficient electron donors, and resulted in a 23.53% increase in denitrification rate compared to the naturally formed biofilm control group [59].

Comparing the two cathodic denitrifying BES pathways, the direct electron transfer pathway operates at very low cell voltages and is energy-neutral or even energy-positive, whereas hydrogenotrophic denitrification requires applied voltages of 1.0–1.5 V but achieves higher nitrate reduction rates of up to 95% removal at 70–130 mg NO_3_^−^–N L^−1^. Relative to the Anammox-based and nitrifying BES pathways, cathodic denitrifying BES is capable of reducing oxidized nitrogen species. Therefore, a cathodic denitrifying BES is most effective when integrated into combined processes, which also represent the most frequently studied combined configurations (Figure 2).

### 2.5. Combined Process

The conversion of NH_4_^+^ to N_2_ can also be achieved by integrating the above-mentioned pathways into one reactor forming combined BESs for nitrogen removal. Zhan et al. simultaneously cultured nitrifying and hydrogenotrophic denitrifying microorganisms using oxygen generated by electrolysis at the anode and hydrogen produced at the cathode, respectively. Even under high dissolved oxygen (DO) conditions (>4 mg/L), an anoxic microenvironment could be maintained within the biofilm, allowing efficient hydrogenotrophic denitrification and complete conversion of NH_4_^+^ to N_2_ within a single reactor [51]. In a hybrid system coupling constructed wetland (CW) with a BES (CW-BES), the CW unit primarily enriched heterotrophic denitrifiers, which utilized organic carbon from the influent for denitrification. The BES unit employed a nickel-foam cathode to generate hydrogen via water electrolysis, providing an electron donor for hydrogenotrophic denitrifiers to reduce NO_3_^−^. Besides providing hydrogen as an electron donor, the electrochemical process generated CO_2_ on the carbon anode, thereby buffering the system pH and maintaining it at 7.5 ± 0.3. The removal efficiency reached 98.11% for NO_3_^−^ and 63.03% for TN [53]. The nitrogen removal rate of the single-chamber stainless steel BES constructed by Song et al. reached 19.68 mg L^−1^ d^−1^. A voltage of 1.5 V was applied with internal circulation to maintain appropriate pH and mass transfer conditions. This facilitated synergistic interactions between the functional microbial communities on the anode and cathode, enabling autotrophic denitrifying bacteria to use electrode-derived electrons or H_2_ as an electron donor to reduce NO_3_^−^/NO_2_^−^ to N_2_ [35].

In combined BESs for nitrogen removal, different microorganisms can also cooperate by sharing electrons, substrates, and electron carriers, collectively accomplishing complex reaction pathways that are difficult for a single microbial group to achieve. For instance, in a system coupling bioelectrochemical technology with sulfur-based autotrophic denitrification (CBSAD), sulfur-based autotrophic denitrifying bacteria used sulfur to reduce NO_3_^−^ to N_2_. The generated H^+^ was transferred to the cathode, where it served as the substrate for hydrogen-autotrophic denitrifying bacteria. This further removed the residual NO_3_^−^ and NO_2_^−^ in the effluent. The total nitrogen removal rate reached 95–100% without NO_3_^−^ and NO_2_^−^ accumulation in the effluent [50]. In a dual-chamber BES, NH_4_^+^ was used as the sole electron donor, with the anode serving as the terminal electron acceptor for NH_4_^+^ oxidation. Nitrifying bacteria oxidized NH_4_^+^ to NO_3_^−^ (accounting for about 95% of the consumed NH_4_^+^-N) via an EET pathway, accompanied by minor NO_2_^−^ production. At the same time, heterotrophic bacteria such as *Empedobacter* used soluble microbial products (SMPs) released by autotrophic bacteria for growth and denitrification [23]. Xu et al. constructed a single-chamber BES, in which the cathode was enriched with diverse functional microbial groups, including heterotrophic denitrifiers, electroactive autotrophic denitrifiers, partial denitrifiers, and Anammox. In this system, heterotrophs used organic matter for denitrification, electroactive autotrophs directly employed cathode electrons to reduce NO_3_^−^/NO_2_^−^, partial denitrifiers converted NO_3_^−^ to NO_2_^−^ for Anammox, and Anammox consumed NH_4_^+^ and NO_2_^−^ to produce N_2_. This configuration enabled simultaneous removal of NO_3_^−^/NO_2_^−^ and NH_4_^+^-N under low C/N ratio conditions [16]. In a dual-chamber BES, an external voltage of 0.7–1.5 V was applied to the anode chamber to drive water electrolysis and oxygen production, providing DO for the oxidation of NH_4_^+^-N by nitrifying bacteria. The NO_3_^−^ generated at the anode was subsequently pumped into the cathode chamber, where autotrophic denitrifiers used electrons supplied by the cathode (directly or via H_2_ mediation) to perform denitrification, ultimately converting NO_3_^−^ to N_2_. This system achieved simultaneous nitrification and denitrification without the need for external organic carbon addition or aeration [37].

The key mechanistic advantage of combined processes is spatial and metabolic division of multiple pathways: anode-zone communities conduct oxidative steps while cathode-zone communities perform reductive steps, creating a self-sustaining nitrogen transformation network within a single reactor. This contrasts with single-pathway BES configurations, where incomplete nitrogen conversion limits overall TN removal, e.g., NO_3_^−^ accumulation in nitrifying BES. However, the superior performance of combined processes comes at the cost of substantially increased operational complexity. This configuration requires the simultaneous management of multiple functional microbial communities, competing electron acceptors, and spatially differentiated redox microenvironments, which necessitates the precise control of voltage, HRT, and substrate loading. The trade-off between performance and controllability is therefore the defining engineering challenge for combined BES processes. This also explains why the highest TN removal efficiencies reported in Table 2 coexist with some of the most variable and least reproducible data across the studies reviewed. Addressing this trade-off through improved reactor design, electrode engineering, and microbial community management is essential for scalable engineering applications.

## 3. Key Factors for Efficient Bioelectrochemical Nitrogen Removal

**Figure 5:**
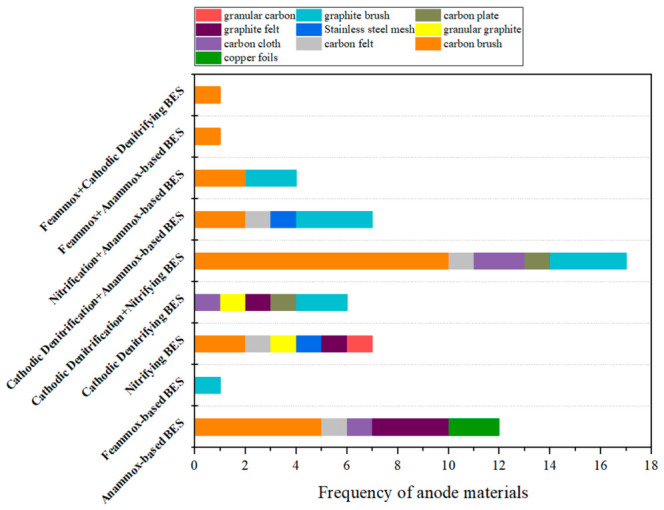
Frequency of anode materials used in BESs for nitrogen removal. Please see Section 3 for the detailed dataset selection criteria.

**Figure 6:**
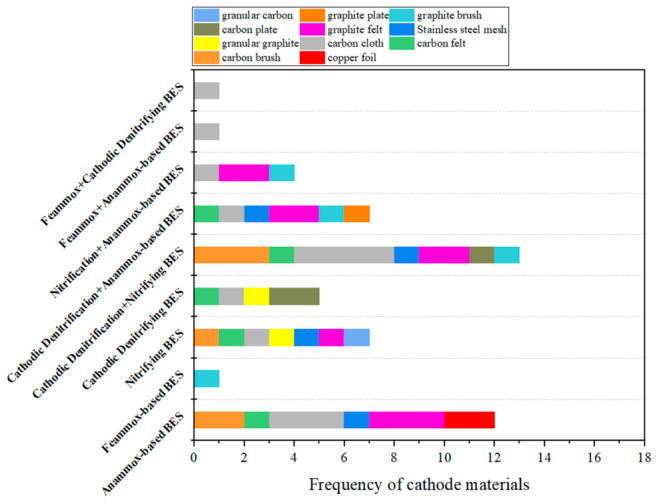
Frequency of cathode materials used in BESs for nitrogen removal. Please see Section 3 for the detailed dataset selection criteria.

To ensure the transparency and reproducibility of the comparative analyses presented in Figure 2, Figure 5 and Figure 6, a systematic literature search is conducted using the Web of Science database, with records updated as of 6 May 2026. The selection criteria are as follows: (1) for single configurations such as Anammox-based BESs, the following keywords and Boolean operators are used: bioelectrochemical nitrogen removal AND single configuration AND (single chamber OR double chamber) NOT other configurations. The keywords used for single configurations included Anammox, nitrification, denitrification, and Feammox. (2) For combined configurations such as cathodic denitrification + nitrifying BESs, the following keywords and Boolean operators are used: bioelectrochemical nitrogen removal AND combined configuration AND (single chamber OR double chamber). The keywords used for combined configurations included nitrification AND denitrification, denitrification AND Anammox, nitrification AND Anammox, Feammox AND Anammox, and Feammox AND denitrification. (3) Reference management software, followed by manual verification, is used to identify and remove duplicate records. (4) Review articles, conference abstracts, book chapters, patents, and non-English publications are excluded. This search and screening process yields 59 eligible entries in total. Each article is then manually reviewed to extract the data used to determine the frequencies of electrode materials shown in Figure 5 and Figure 6.

It is important to clarify that the frequencies reported in Figure 2, Figure 5 and Figure 6 reflect current research trends, i.e., the number of published studies that have adopted each configuration or electrode material. These frequencies do not directly represent engineering relevance, commercial viability, or large-scale applicability. A high frequency in the literature indicates that a particular configuration or material has attracted substantial research interest, but this does not necessarily imply superior practical performance.

The research progress for the nitrogen removal in BES is summarized in Table 2. Most of the studies remained at laboratory scale and utilized synthetic wastewater with simplified, ideal compositions. Though impressive nitrogen removal performance was achieved in some cases, e.g., 99% nitrogen removal in a single-chamber BES with combined anodic nitrification and cathodic denitrification [15], the overall nitrogen removal rates were generally low, typically ranging from 8.27 to 56 g N m^−3^ d^−1^. This performance was not comparable with the removal rates of 80–1360 g N m^−3^ d^−1^ for conventional heterotrophic denitrification. Additionally, the data in Table 2 also indicate considerable instability in nitrogen removal performance, with the fluctuating nitrogen removal efficiencies of 12–99% and the nitrogen removal rates of 7.5–33.38 g N m^−3^ d^−1^, or even 12–56 g N m^−3^ d^−1^ in some cases.

The two limitations observed across BES studies, low nitrogen removal rates and long-term instability, are the results of intrinsic mechanistic constraints common to current BES configurations. Three interconnected mechanistic bottlenecks underlie both limitations. First, EET is inherently rate-limiting. Whether electron delivery occurs through direct electrode-microbe interactions or H_2_-mediated transfer, nitrogen conversion remains constrained by interfacial electron exchange, which is far slower than intracellular metabolism. In thick biofilms, cells located away from the electrode become electrochemically disconnected, contributing to biomass accumulation more than to nitrogen removal and thereby lowering volumetric performance. Second, the key functional microorganisms in BESs often grow slowly and recover poorly from disturbance. This is especially true for Anammox bacteria, whose long doubling time makes the system highly vulnerable to voltage fluctuations, substrate shocks, oxygen intrusion, and other perturbations, leading to prolonged recovery periods and operational instability. Third, strong competition among co-existing microbial groups can divert electrons, substrates, and attachment sites away from the intended nitrogen removal pathway. In combined processes, nitrifiers, Anammox bacteria, denitrifiers, and electroactive organisms compete dynamically, and their relative abundances shift with operating conditions. Together, these mechanisms explain why low removal rates and instability frequently co-occur across BES configurations and provide the mechanistic context for evaluating substrate concentration, applied voltage, electrode material, and microbial interactions in the following sections.

This suggests a substantial gap between the nitrogen removal performance and various operational parameters such as reactor configuration, applied voltage, inoculum source, electrode materials, and influent substrate concentration, etc. Thus, this section critically examines the influence of substrate concentration, applied voltage, electrode materials, and microbial interactions, which may be the key factors and bottlenecks that undermine system performance and robustness.

### 3.1. Substrate Concentration

Appropriate substrate concentrations enhance nitrogen removal efficiency by optimizing the enzyme activity and related gene expression of functional microorganisms, whereas imbalanced substrate levels inhibit key enzymatic activities and disrupt gene expression in these microbes. In Anammox-based processes, when NH_4_^+^-N was 50–100 mg/L, NO_2_^−^-N was 25–50 mg/L, and the NO_2_^−^/NH_4_^+^ ratio was 1.3–1.5, Anammox efficiently expressed hydrazine synthase via the hzsA gene, achieving a TN removal rate of 82.6–99.1% [39]. However, if the NH_4_^+^-N concentration increased beyond 200 mg/L and the NO_2_^−^/NH_4_^+^ ratio dropped below 0.2, the expression of the hzsA gene was downregulated by approximately 40%, inhibiting hydrazine synthase activity and leading to insufficient electron acceptors, which reduced the TN removal rate to 38.6–70.3% [15,30,60]. High NH_4_^+^-N concentrations also inhibit the nitrification process. Significant influences of ammonia on microbial community were observed in both anode and cathode biofilm [61]. In a single-chamber BES for nitrogen removal, when NH_4_^+^-N increased from 50 mg L^−1^ to 200 mg L^−1^, the removal efficiency dropped from 98.7 to 70.3% (Table 2). The reason is an excessively high concentration reduced the cell membrane permeability of nitrifying bacteria and inhibited the activity of ammonia monooxygenase [15,35]. Thus, high DO levels are needed to enhance enzymatic reaction rates of nitrifying bacteria, thereby mitigating inhibition and indirectly sustaining the subsequent denitrification performance [62].

Substrate concentration also affects metabolic interaction between microorganisms. Under high ammonia load, the selective pressure narrows the microbial diversity. This pressure may facilitate the proliferation of bacteria that are tolerant to ammonia or have specific functions, while inhibiting more sensitive species [63]. In mixed microbial communities, competition for NH_4_^+^-N exists between AOB and Anammox. At an NH_4_^+^-N concentration of 50 mg/L, AOB preferentially used NH_4_^+^-N, causing the abundance of Anammox to decrease from 32.8% to 5.7%, further deteriorating the decline in nitrogen removal efficiency [30]. AOB and AOA in nitrification processes maintained a stable removal rate of 87.8–99% by efficiently synthesizing ammonia monooxygenase at NH_4_^+^-N concentrations of 20–100 mg/L [1].

Furthermore, substrate concentration regulates the accumulation and transformation of intermediates. Abundant electron donors are beneficial for alleviating the accumulation of NO_2_^−^ [64]. In a hydrogenotrophic denitrification BES enhanced by cathode surface modification, when NO_3_^−^-N concentration was 70–130 mg/L, denitrifiers can efficiently reduce NO_3_^−^ to N_2_. This process avoids the accumulation of intermediates, achieving a NO_3_^−^ removal rate of up to 95% [65]. An increase in NO_3_^−^ concentration from 12 mg-N/L to 78 mg-N/L boosted cathodic reduction and NO_2_^−^ production, thereby supplying additional NO_2_^−^ for the coupling Anammox bacteria in the system [66]. Once NO_3_^−^ levels exceeded 130 mg/L, however, the denitrification rate surpassed the electron transfer rate, causing the accumulation of toxic NO, severely impairing denitrification efficiency [65]. Thus, appropriate substrate concentrations optimize microbial metabolism and enhance nitrogen removal efficiency, while imbalanced substrate levels suppress enzymatic activity, disrupt microbial balance, and lead to the accumulation of toxic intermediates.

Overall, substrate concentration defines the boundary between pathway enhancement and inhibition. An appropriate NH_4_^+^-N concentration promotes functional gene expression and supports total nitrogen removal, whereas excessive concentrations can inhibit functional microorganisms and reduce nitrogen removal performance. It is worth noting that the substrate concentration effects reviewed above were characterized under synthetic wastewater conditions using single nitrogen species at defined concentrations (Table 2). In real wastewater, nitrogen species coexist with variable organic carbon, competing ions, and inhibitory compounds, creating substrate interactions that are absent from laboratory studies. Therefore, the results reported in the literature are derived from conditions that systematically underestimate the operational challenges of practical deployment. This represents a critical gap between current BES research and large-scale application.

### 3.2. Applied Voltage

**Figure 7:**
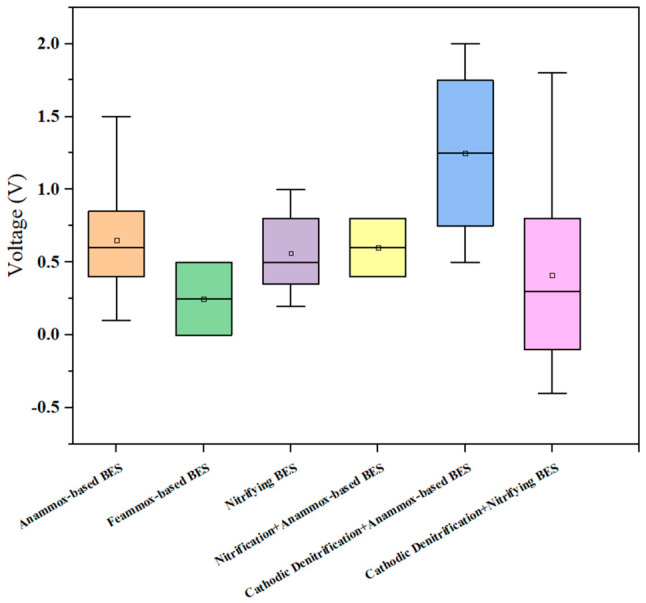
Operating voltage used in BESs for nitrogen removal.

Voltage plays a key role in optimizing removal pathways and enriching functional microbial consortia by directly driving electron transfer and influencing microbial metabolism, thereby enabling an efficient and controllable bioelectrochemical denitrification process. Appropriate voltage can compensate for the electron demand under high substrate concentrations and promote the EET process by activating the expression of multi-heme cytochromes in Anammox. Applying a certain voltage simultaneously can reduce the inhibition of some harmful substances on the activity of microorganisms [67]. Van Khanh et al. discovered that altering the cathode voltage would affect the enrichment and distribution of microorganisms and prevent the accumulation of nitrous oxide [68]. However, Figure 7 shows that the optimal operating voltage ranges varied among different BES. For instance, in an anodic nitrification–cathodic denitrification BES, increasing the operating voltage from 0.2 V to 0.4 V significantly enhanced the EET efficiency between the electrode and microorganisms and the NH_4_^+^-N removal efficiency from 70.3% to 92.6% [15]. In an anodic NH_4_^+^ oxidation process, a constant voltage of 0.6 V effectively reduced the electron-transfer stress induced by a high NH_4_^+^-N concentration of 280 mg/L, simultaneously suppressing the overgrowth of heterotrophic microorganisms and reducing NO_2_^−^ accumulation, which improved the NH_4_^+^-N removal rate from 41.3% to 55.9% [36].

Data presented in Figure 7 and Table 2 indicate that a voltage range of 0.3–0.8 V was used in most BES studies for nitrogen removal, while in some cases, voltage as high as 2.0 V was also investigated. In a dual-chamber BES operated at 1.5 V, the supply of oxygen required for nitrification and hydrogen required for denitrification was effectively maintained, achieving a TN removal efficiency of 85% [37]. Yang et al. found that at an applied voltage of 1.0 V, the electric field not only drove ion migration to promote the directional transport of reactants including NH_4_^+^, but also significantly enhanced the activity of nitrifying functional bacteria such as *Nitrospira*, enabling the system to achieve a TN removal efficiency of 61.40% [40]. Applying voltage can induce the electroactivity of AOB, regulating the abundance of the key functional genes including hydroxylamine oxidation (hao), nitrate reduction (narG, narH, narI, napA, napB), nitrite reduction (nirK, nirS), and nitric oxide reduction (norB, norC) [41]. For the Anammox process, voltages of 0.4–1.3 V can activate the expression of multi-heme cytochromes in Anammox, up-regulating hydrazine synthase activity by 40–50%. Additionally, studies have shown that for marine Anammox in a single-chamber BES, a driving voltage of 1.1 V promoted the formation of anode biofilms. The microbial community diversity on the anode surface was higher than that in the suspended bulk [28,29].

However, once the voltage exceeds the tolerance threshold, significant inhibition occurs to the microorganisms, including cell membrane damage and denaturation of EET-associated proteins. High voltage has a particularly significant inhibitory effect on non-electrochemical HDB, and causes the re-oxidation of nitrite at the anode [69]. In Anammox processes, when the voltage surpassed 0.8 V (vs. Ag/AgCl), cell membrane permeability increased by 3–5 times, accompanied by leakage of key enzymes and denaturation of EET-related proteins, leading to a drastic decline in denitrification efficiency to 0% [32]. Furthermore, inappropriate voltage can divert electron flow toward competitive side reactions. In a single-chamber nitrification–denitrification BES operated at 1.8 V, excessive oxygen generation at the anode caused electrons to be preferentially utilized for oxygen reduction rather than NO_3_^−^ reduction, reducing the TN removal rate from 19.68 mg L^−1^ d^−1^ to 8.27 mg L^−1^ d^−1^ [35]. Therefore, the applied voltage should be carefully optimized to prevent inhibitory effects on functional microorganisms and to direct the reaction toward the desired pathway.

A narrow voltage range is used in BES for nitrogen removal (Figure 7), but there is only a small margin between this range and the voltage threshold that may lead to cell membrane damage, enzyme denaturation, competitive side reactions, and deteriorated performance (Table 2). This means that uncontrolled voltage fluctuations, due to power supply instability or changes in solution conductivity, can shift the system from performance enhancement into active inhibition. The practical implication is that long-term voltage stability is as important as the absolute voltage value, yet very few of the studies in Table 2 report the voltage stability over extended operation periods. Further investigation is therefore needed to pave the way towards practical applications.

### 3.3. Electrode Materials

In BESs for nitrogen removal, the specific surface area, conductivity, and stability of electrode materials effectively influence microbial attachment, electron transfer, and eventually the nitrogen removal performance. Sanchis-Carbonell et al. increased the theoretical surface area by constructing these pyramid-based electrodes, achieving a better nitrogen removal effect [70]. Carbon-based materials are the most commonly used for both anodes and cathodes (Figure 5 and Figure 6). In an Anammox-based BES operated at 0.8 V without exogenous NO_2_^−^, a carbon-brush electrode with a high specific surface area (1000–2000 m^2^/g) provided sufficient attachment sites for microorganisms, ensuring a high abundance of Anammox bacteria. Simultaneously, its favorable conductivity promoted EET, achieving an NH_4_^+^-N removal efficiency of 41% and a coulombic efficiency of 40.92% [38]. In contrast, titanium mesh, due to its limited specific surface area (<100 m^2^/g) and the formation of insulating TiO_2_ layer, severely inhibited microbial attachment under high voltage and electron transfer, leading to performance failure under high load conditions [32]. Titanium anodes need to be coated with ruthenium dioxide in order to stabilize their surface and molecular structure and reduce operating costs [71]. For hydrogenotrophic denitrification-dominated processes, polypyrrole (PPy) modified carbon felt significantly promoted NO_3_^−^ reduction by enhancing electrode conductivity and reducing electron transfer resistance, achieving a denitrification rate of 95% at a NO_3_^−^ concentration of 70–130 mg L^−1^ [65].

Electrode materials also shape the microenvironment by affecting interfacial mass transfer, potential distribution, and biofilm architecture. The electrode materials, such as biochar, have a strong interaction with NO_3_^−^, which can undergo a normal adsorption process and promote nitrogen removal [72]. In a simultaneous nitrification–denitrification BES equipped with high porosity carbon felt electrodes, aerobic microenvironments were created in an anode zone and anoxic microenvironments in a cathode zone, thus successfully enriched nitrifying bacteria and denitrifying bacteria in the anode and cathode zones, respectively. In this study, a TN removal rate of 19.68 mg L^−1^ d^−1^ was achieved at a voltage of 1.5 V [35]. In partial nitritation-Anammox processes, carbon brush electrodes concurrently satisfied the attachment requirements of AOB and provided an anaerobic metabolic interface for Anammox, achieving an NH_4_^+^-N removal rate of 68.12% at 0.4 V [31].

Moreover, during long-term operation or under harsh conditions, the chemical stability and biocompatibility of electrode materials become critical limiting factors. Carbon-based materials remain stable in neutral to weakly alkaline environments without toxic leaching, enabling Anammox consortia to maintain a high abundance of 32.8% over 150 days [30]. The carbon surface is easily regenerable, and a relatively small electrode surface area is sufficient to meet the requirements of electron transfer [73]. In contrast, metal-based materials such as stainless steel and titanium mesh, are susceptible to chloride ion corrosion or surface oxidation under high salinity or high voltage conditions. This not only increases electron transfer resistance but also inhibits microbial activity due to the leaching of dissolved metals or the detachment of oxide layers. Consequently, these materials are primarily suitable for specific scenarios with low loads and short operation cycles [29,32]. In conclusion, electrode materials are critical to the efficiency of BESs for nitrogen removal, determining biocompatibility and electron transfer efficiency. Carbon-based materials are commonly used for both anode and cathode, but in order to improve the stability and removal rate of BESs for nitrogen removal, further research is still needed to develop more conductive and biocompatible electrodes to support robust electroactive biofilms and to enhance the nitrogen removal efficiency of BESs.

Carbon-based materials offer high biocompatibility and chemical stability and are most commonly used for both anodes and cathodes (Figure 5 and Figure 6). However, they suffer from limited conductivity and poor mechanical durability under long-term operation. Metal-based and modified materials provide superior conductivity but introduce corrosion, toxicity, and cost barriers that challenge their sustained use in complex wastewater environments. Developing electrode material that simultaneously satisfies all requirements, including high specific surface area, high conductivity, long-term chemical stability, biological compatibility, and low cost, is therefore challenging. This materials gap is among the most significant engineering barriers to scaling up BESs for nitrogen removal.

### 3.4. Interactions of Functional Microorganisms

The functional microorganisms in BESs mainly interact with each other in two ways: interspecies electron transfer and complementary metabolisms. The former relies on EET to drive redox reactions, while the latter optimizes nitrogen removal pathways via substrate exchange among functional microorganisms. The interspecies electron transfer between syntrophic partners makes it possible for the microorganism to overcome energy barriers and accomplish certain metabolic processes [74]. Applied voltage has been shown to promote the secretion of extracellular polymeric substances (EPS) and c-type cytochromes, enhancing the EET capability of functional bacteria like Anammox [38]. This enables electron transfer from electron-donating Anammox (e.g., *Candidatus Brocadia*) to coexisting AOB or other electron acceptors, thereby achieving NH_4_^+^ oxidation under NO_2_^−^ deficient conditions [31]. Furthermore, microorganisms can also perform interspecies electron transfer via metabolic intermediates. In sulfur–iron mediated autotrophic denitrification systems, the addition of ferrous ions (Fe^2+^) promoted interspecies electron transfer with polysulfides serving as diffusive electron carriers. This process enriched taxa with strong electron transfer capacities, such as *Sulfurimonas*, and upregulated the expression of functional genes.

Furthermore, in BESs, functional microorganisms cooperate with each other through complementary metabolisms to achieve nitrogen removal. In a BES for nitrogen removal, AOB *Nitrosomonas* oxidized NH_4_^+^ to NO_2_^−^ driven by electrode potential, which was then reduced by coexisting Anammox bacteria *Candidatus Brocadia* and heterotrophic denitrifying bacteria, achieving complete conversion of NH_4_^+^ to N_2_ through synergistic metabolism [24]. In a BES integrating Anammox with hydrogenotrophic denitrification, Anammox bacteria *Candidatus Kuenenia* dominated the removal of NH_4_^+^ and NO_2_^−^. At the same time, the NO_3_^−^ produced was reduced to N_2_ by hydrogenotrophic denitrifying bacteria, such as *Hydrogenophilales*, attached to the cathode, using hydrogen generated from water electrolysis. These two processes complement each other spatially and functionally, achieving a TN removal rate as high as 99.1% [39]. When stochastic community assembly dominated, higher community diversity led to declining functional efficiencies in EET [75]. Thus, in BESs for nitrogen removal from wastewater with low C/N ratios, it is feasible to simultaneously enrich heterotrophic denitrifiers, hydrogenotrophic denitrifiers, and Anammox by regulating voltage and hydraulic conditions [16]. In this system, heterotrophs are responsible for removing residual organic matter, hydrogen generated at the cathode drives hydrogenotrophic denitrifiers to reduce NO_3_^−^, and Anammox bacteria convert part of the NH_4_^+^ and NO_2_^−^ to N_2_, achieving efficient and stable nitrogen removal performance through complementary interactions [33,34].

Overall, the syntrophic and electron-sharing interactions that enable high-efficiency nitrogen removal in well-functioning combined systems are also the primary source of instability when those interactions are disrupted. This fragility may lead to reduced nitrogen removal rates and long-term instability, but it is difficult to overcome as the functional relationships between Anammox, nitrifiers, heterotrophic denitrifiers, and electroactive bacteria are dynamic, transient, and difficult to maintain. Another challenge is that BES communities are shaped by strong electrochemical selection pressure, which simultaneously enriches the desired functional microorganisms and reduces overall community diversity. As a result, the system has less functional redundancy, lower resilience to perturbation, and poorer long-term stability. Given the complexity of these microbial interactions, most current evidence still comes from laboratory studies under idealized conditions. Therefore, long-term studies using real wastewater and culture-independent multi-omics approaches are needed to clarify microbial interaction mechanisms under practical conditions and to support the rational design of BESs at engineering scale.

## 4. Outlook

The critical analysis in this review identifies low nitrogen removal rate and long-term operational instability as the two central engineering barriers preventing BES nitrogen removal from being applied practically. Addressing these requires not only operational parameter adjustment but targeted progress in reactor design, electrode materials, microbial community engineering, and real-wastewater process validation. Concrete research directions in each domain are outlined below:

(1) The dominant use of single-chamber configurations (Figure 2), while experimentally convenient, creates inherent electrochemical conflicts between anodic and cathodic microenvironments that suppress simultaneous nitrification and denitrification performance. Future reactor design should prioritize structured spatial separation of functional zones. Besides the double chamber configuration, another possible direction is a modular multi-stage BES, which also enables targeted replacement or maintenance of individual stages without disrupting the entire system. For example, an upstream nitrifying BES module feeds a downstream Anammox-BES or cathodic denitrifying BES module, decoupling the conflicting optimal voltage for each process and allowing independent control of each stage.
(2) The materials gap is one of the most important engineering bottlenecks for scale-up, because electrode materials must combine high specific surface area, high conductivity, long-term chemical stability, biocompatibility, and low cost. Carbon-based materials are the most widely used. Among them, electroconductive biochar is particularly attractive as a low-cost, scalable electrode material. Biochar offers inherent surface functional groups that favor microbial attachment, and its feedstock is an abundant by-product of wastewater treatment and the agricultural sector. In addition, its conductivity can be further enhanced by controlling pyrolysis conditions and introducing metal oxide dopants, without the biotoxicity concerns associated with noble-metal coatings.
(3) Current understanding of the microbial interactions in BES is largely correlative because community composition is usually characterized at specific time points and correlated with performance, but the mechanisms governing community assembly, stability, and functional redundancy remain poorly resolved. To address this issue, time-resolved metatranscriptomics can be used to determine actively expressed genes under different conditions, bridging genomic potential and metabolic activity; stable isotope probing combined with nanoscale secondary ion mass spectrometry can be used to resolve biofilm activity at single-cell resolution and define the effective EET zone. In addition, functionally defined consortia of Anammox, nitrifiers, heterotrophic denitrifiers, and electroactive bacteria rather than complex mixed sludge, should be constructed and systematically tested. Amplicon sequencing can be used to track the relative abundances of functional taxa over extended operation and to evaluate the consortium stability.
(4) Translating BES nitrogen removal to real wastewater requires a structured research program that moves systematically through three stages: (i) synthetic wastewater supplemented with real-matrix components, such as humic substances, to isolate the effect of individual matrix components on BES performance; (ii) long-term continuous-flow tests with real municipal or industrial wastewater to assess performance stability, electrode degradation, and community dynamics; and (iii) pilot-scale validation at wastewater treatment facilities to quantify scale-up penalties, current non-uniformity, and temperature and pH gradient effects that are absent at laboratory scale but critical for engineering design.

## 5. Conclusions

This review critically synthesized the mechanisms, performance, and operational parameters of five BES nitrogen removal pathways, including Anammox-based BES, Feammox-based BES, nitrifying BES, cathodic denitrifying BES, and combined processes. The following key findings were identified: (1) a persistent performance gap exists between laboratory-scale BES and conventional technologies. Most BES studies report nitrogen removal rates of 8.27–56 g N m^−3^ d^−1^, which are substantially lower than conventional heterotrophic denitrification (80–1360 g N m^−3^ d^−1^) and conventional Anammox (up to 76.7 kg N m^−3^ d^−1^). In addition, long-term operational instability is universal and mechanistically rooted, with nitrogen removal efficiencies fluctuating between 12% and 99% (Table 2). These two limitations arise from the sensitivity of BESs to perturbations, which is driven by rate-limiting EET kinetics at the electrode-biofilm interface, the slow growth of key functional microorganisms, and complex microbial interactions. (2) Substrate concentration defines the boundary between pathway enhancement and inhibition. An appropriate NH_4_^+^-N concentration promotes functional gene expression and supports total nitrogen removal, whereas excessive concentrations can inhibit functional microorganisms and reduce nitrogen removal performance. (3) An applied voltage of 0.3–0.8 V represents the typical operating window for most BES configurations, although the optimal range varies by configuration. However, the margin between this window and the voltage threshold at which performance begins to deteriorate is narrow. This means that long-term voltage stability is as important as the absolute voltage value for engineering-scale applications. (4) Carbon-based materials are the most commonly used for both anodes and cathodes, but suffer from limited conductivity and poor mechanical durability under long-term operation. The development of electrode materials with low cost, scalability, and long-term stability remains the most significant engineering bottleneck for scale-up. (5) BES for nitrogen removal has the conceptual and mechanistic potential to become a sustainable, low-carbon alternative for wastewater nitrogen removal. Realizing this potential requires the transition from demonstrating performance under idealized conditions to systematically addressing the rate, stability, and scalability barriers identified in this review.

## Figures and Tables

**Table 1:** Summary of various BESs for nitrogen removal.

Process	Reaction	Potential (V vs. SHE)	Microorganism(s)	Growth Rate (d^−1^)	Ks [S]	Ks [EA]	Ref.
EET-dependent Anammox	(O) 2NH_4_^+^→N_2_ + 8H^+^ + 6e^−^ (B) NH_4_^+^ + H_2_O→NH_2_OH + 3H^+^ + 2e^−^ (B) NH_4_^+^ + NH_2_OH→N_2_H_4_ + H_2_O + H^+^ (B) N_2_H_4_→N_2_ + 4H^+^ + 4e^−^	0.6	*Candidatus Kuenenia stuttgartiensis*; *Candidatus Brocadia fulgida*; *Candidatus Scalindua profunda*; *Candidatus Jettenia caeni*	4–14	<5	<5	[1]
Feammox based BES	(O) 3Fe_2_O_3_ + 10H^+^ + 2NH_4_^+^→6Fe^2+^ + 9H_2_O + N_2_			<1	0.6		[22]
	(O) 3Fe_2_O_3_ + 10H^+^ + NH_4_^+^→6Fe^2+^ + 7H_2_O + NO_2_^−^ (O) 4Fe_2_O_3_ + 14H^+^ + NH_4_^+^→8Fe^2+^ + 9H_2_O + NO_3_^−^		*Acidimicrobiaceae* sp. A6				
Nitrifying BES	(O) NH_4_^+^ + 3H_2_O→NO_3_^−^ + 10H^+^ + 8e^−^ (O) NH_4_^+^ + 2H_2_O→NO_2_^−^ + 8H^+^ + 6e^−^ (B) NH_3_ + O_2_ + 2H^+^ + 2e^−^→NH_2_OH + H_2_O (B) NH_2_OH + H_2_O − 4e^−^→NO_2_^−^ + 5H^+^ (B) NO_2_^−^ + H_2_O − 2e^−^→NO_3_^−^ + 2H^+^ (R) NH_4_^+^ + H_2_O→NH_2_OH + 3H^+^ + 2e^−^ (R) NH_2_OH + H_2_O→NO_2_^−^ + 5H^+^ + 4e^−^ (R) NO_2_^−^ + H_2_O→NO_3_^−^ + 2H^+^ + 2e^−^	0.883 0.074 0.36 0.44 0.73 0.375	Ammonia-oxidizing bacteria (AOB); Ammonia-oxidizing archaea (AOA); *Candidatus Nitrosopumilus maritimus*; *Candidatus Nitrosocaldus yellowstonii*; *Candidatus Nitrosotalea devanaterra*; *Candidatus Nitrosocaldus islandicus*; Nitrite-oxidizing bacteria (NOB); *Nitrococcus mobilis*; *Nitrospirae*; *Candidatus Nitrospira defluvii*; *Nitrospinae*; *Nitrospina gracilis*; *Nitrolancetus hollandicus*; *Nitrosomonas*	<1 <5	0.8–12 5–44 9–544	1–15 22–166	[23,24,25]
Cathodic denitrifying BES	(O) NO_3_^−^ + 5H_2_→N_2_ + 2OH^−^ + 4H_2_O (O) NO_2_^−^ + 3H_2_→N_2_ + 2OH^−^ + 2H_2_O (R) NO_3_^−^ + 5e^−^ + 6H^+^→ ^1^/_2_ N_2_ + 3H_2_O (R) NO_3_^−^ + 2e^−^ + 2H^+^→NO_2_^−^ + H_2_O (R) NO_2_^−^ + e^−^ + 2H^+^→NO + H_2_O (R) NO + e^−^ + H^+^→ ^1^/_2_N_2_O + ^1^/_2_H_2_O (R) ^1^/_2_N_2_O + e^−^ + H^+^→ ^1^/_2_N_2_ + ^1^/_2_H_2_O	0.74 0.375 0.42 1.175 1.355	*Shewanella* *Pseudomonas* *Alcaligenes* *Paracoccus*				[24,26,27]

Notes: (1) Labels before each reaction: (O) = Overall stoichiometric microbial conversion; (B) = biochemical pathway step catalyzed by enzyme; (R) = simplified redox reaction. (2) Potential (V vs. SHE): electrode potential (volt versus standard hydrogen electrode). (3) EET: extracellular electron transfer. (4) Growth rate: specific growth rate. (5) BESs: bioelectrochemical systems. (6) Ks [S]: substrate saturation constant. (7) Ks [EA]: electron acceptor saturation constant.

**Table 2:** Application of BESs for nitrogen removal.

Reactor Design	Voltages	Inoculation Source	Anode Materials	Cathode Materials	Influent (mg/L)	Nitrogen Removal	CE (%)	Ref.
Single-chamber BES	−0.1, 0, 0.1, 0.2, 0.3, 0.4, 0.5, 0.6 V (vs. SHE)	*Ca. Brocadia* *Ca. Scalindua*	Graphite rod	Platinum mesh	NH_4_^+^-N: 20, 80, 100; NO_2_^−^-N: 34.5		87.8 ± 3.2%	[1]
Double-chamber BES	0.6 V (vs. SHE)	*K. stuttgartiensis*	Carbon cloth	Platinum mesh	NH_4_^+^-N: 100			[1]
Single-chamber BES	0.2, 0.3, 0.4 V (vs. Ag/AgCl)	Anode: nitrifying inoculum Cathode: denitrifying inoculum	Carbon felt	Carbon felt	NH_4_^+^-N: 50, 100, 150, 200	NH_4_^+^-N = 50 mg/L 0.2 V: 98.7%; NH_4_^+^-N = 100 mg/L 0.2 V: 99%; NH_4_^+^-N = 150 mg/L 0.2 V: 77.2%; NH_4_^+^-N = 200 mg/L 0.2 V: 70.3%; NH_4_^+^-N = 200 mg/L 0.3 V: 87.9%; NH_4_^+^-N = 200 mg/L 0.4 V: 92.6%;	63.9% 56.8% 70.4% 82% 89.5% 94.4%	[15]
Single-chamber BES	0.2 V (total applied voltage)	Active sludge	Graphite rod	Carbon felt	NH_4_^+^-N: 200, 150, 100, 0; NO_2_^−^-N: 250, 0; NO_3_^−^-N: 500, 300, 0; COD: 2000, 1000, 0;	COD/N = 2: 695.6 g N m^−3^ d^−1^ COD/N = 1: 514.5 g N m^−3^ d^−1^	98.16%	[16]
Double-chamber BES		Fresh water sediments	Graphite plate	Graphite plate	NH_4_^+^-N: 90		32.7 ± 7.7%	[23]
Double-chamber BES	0.8 V (vs. SHE)	Anode: aerobic nitrifying reactor and partial nitrifying reactor Cathode: denitrifying BES	Granular graphite	Granular graphite	NH_4_^+^-N: 100	35 ± 10 g N m^−3^ d^−1^	35 ± 13%	[24]
Single-chamber BES	0.4, 0.7, 1 V (vs. Ag/AgCl)	Anammox	Carbon cloth	Carbon cloth	NH_4_^+^-N: 60; NO_2_^−^-N: 80	Step-up voltage: 84%		[28]
Single-chamber BES	0, 0.5, 0.7, 0.9, 1.1, 1.3, 1.5 V	Marine Anammox bacteria (MAB)	Carbon brush	Carbon brush	NH_4_^+^-N: 100	0.5 V: 12 g N m^−3^ d^−1^; 0.7 V: 20 g N m^−3^ d^−1^; 0.9 V: 18 g N m^−3^ d^−1^; 1.1 V: 55 g N m^−3^ d^−1^; 1.3 V: 56 g N m^−3^ d^−1^; 1.5 V: 34 g N m^−3^ d^−1^		[29]
Single-chamber BES	0.6 V (vs. SHE)	Anammox	Carbon brush	Platinum mesh	NH_4_^+^-N: 50, 60, 80, 100; NO_2_^−^-N: 50, 40, 20, 0	NH_4_^+^ = 50 mg/L, NO_2_^−^ = 50 mg/L: 82.6%; NH_4_^+^ = 60 mg/L, NO_2_^−^ = 40 mg/L: 82.6%; NH_4_^+^ = 80 mg/L, NO_2_^−^ = 20 mg/L: 79.4%; NH_4_^+^ = 100 mg/L, NO_2_^−^ = 0 mg/L: 38.6%, 20 mg NH_4_^+^-N L^−1^ d^−1^		[30]
Single-chamber BES	0.4 V (vs. Ag/AgCl)	Partial nitrification + Anammox	Carbon brush	Carbon brush	NH_4_^+^-N: 500; NO_2_^−^-N: 500, 250, 0	NH_4_^+^/NO_2_^−^ = 1:68.12%; NH_4_^+^/NO_2_^−^ = 0.5:64.22%; NH_4_^+^/NO_2_^−^ = 0:57.86%	75.81% 106.53% 173.35%	[31]
Single-chamber BES	0.3, 0.5, 0.8 V (vs. Ag/AgCl)	Anammox	Carbon felt	Titanium mesh	NH_4_^+^-N: 50	0.3 V: 88%; 0.5 V: 48.7%; 0.8 V: 0%		[32]
Single-chamber BES	−0.4, −0.3, −0.2, −0.1, 0.1, 0.2, 0.3, 0.4 V (vs. Ag/AgCl)	Nitrification and denitrification mixture	Carbon felt	Stainless steel mesh	NH_4_^+^-N: 200; COD: 100	−0.4 V: 32.88%, 7.5 g NH_4_^+^-N m^−3^ d^−1^; −0.3 V: 44.1%, 9.23 g NH_4_^+^-N m^−3^ d^−1^; −0.2 V: 50.81%, 33.38 g NH_4_^+^-N m^−3^ d^−1^; −0.1 V: 25.82%, 14.25 g NH_4_^+^-N m^−3^ d^−1^; 0.1 V: 23.7%, 7.98 g NH_4_^+^-N m^−3^ d^−1^; 0.2 V: 40.44%, 24.63 g NH_4_^+^-N m^−3^ d^−1^; 0.3 V: 31.23%, 7.85 g NH_4_^+^-N m^−3^ d^−1^; 0.4 V: 24.22%, 4.88 g NH_4_^+^-N m^−3^ d^−1^	3.17% 16.24% 2.28% 26.74% 30.84%	[33]
Single-chamber BES	0.2, 0.3, 0.4, 0.5 V (vs. Ag/AgCl)	Nitrifying reactor	Carbon felt	Stainless steel tank	NH_4_^+^-N: 100; COD: 100	0.3 V: 32.90 ± 3.39%		[34]
Single-chamber BES	1.2, 1.5, 1.8 V (vs. Ag/AgCl)	Nitrification reactor Denitrification reactor	Carbon brush	Stainless steel	NH_4_^+^-N: 600	1.2 V: 16.05 ± 0.01 g N m^−3^ d^−1^ 1.5 V: 19.68 ± 0.02 g N m^−3^ d^−1^ 1.8 V: 8.27 ± 0.00 g N m^−3^ d^−1^	37.25% 67.86% 88.63%	[35]
Double-chamber BES	0.6 V (vs. Ag/AgCl)	Anode: sewage treatment plant sludge	Carbon felt	Carbon felt	NH_4_^+^-N: 140, 280, 420	NH_4_^+^ = 140 mg/L: 41.3 ± 3.2% NH_4_^+^ = 280 mg/L: 55.9 ± 5.5% NH_4_^+^ = 420 mg/L: 47.7 ± 3.0%	79.6 ± 2.0% 43.5 ± 1.5% 53.4 ± 3.6%	[36]
Double-chamber BES	0.7, 1, 1.5 V (total applied voltage)	Anode: sewage treatment plant sludge Cathode: sewage treatment plant sludge	Carbon brush	Carbon brush	NH_4_^+^-N: 30, 50, 150	Batch mode: 0.7 V: 87.05% 1 V: 94.11% 1.5 V: 96.8% Continuous mode: 1.5 V, NH_4_^+^ = 50 mg/L: 170 g N m^−3^ d^−1^		[37]
Single-chamber BES	0.8 V (total applied voltage)	PN and Anammox reactor sludge	Carbon brush	Carbon brush	NH_4_^+^-N: 100–270	Three carbon brushes: 41%	40.92%	[38]
Single-chamber BES	0.5, 1, 1.5, 2 V (total applied voltage)	Anammox and autotrophic denitrification sludge	Carbon felt	Graphite rod	NH_4_^+^-N: 100, 120, 140; NO_2_^−^-N: 132, 156, 210	0.5 V, NO_2_^−^/NH_4_^+^ = 1.3: 570 g N m^−3^ d^−1^; 1 V, NO_2_^−^/NH_4_^+^ = 1.3: 660 g N m^−3^ d^−1^; 1.5 V, NO_2_^−^/NH_4_^+^ = 1.5: 99.1% 1380 g N m^−3^ d^−1^;		[39]
Double-chamber BES	0.2, 0.6, 1 V (vs. Ag/AgCl)	Anode: anoxic sludge Cathode: aerobic sludge	Titanium mesh	Carbon brush	NH_4_^+^-N: 80; NO_2_^−^-N: 70; NO_3_^−^-N: 70; COD: 300, 400, 500, 600	1 V: 61.40 ± 19.58%		[40]
Single-chamber BES	0.5, 0.7, 0.9, 1.1 V (vs. Ag/AgCl)	Nitrification reactor’s inoculation	Carbon felt	Stainless steel	NH_4_^+^-N: 500; COD: 1000	0.5 V: 67.87 ± 0.25%; 0.9 V: 59.79 ± 1.59%		[41]

Notes: (1) BESs represents bioelectrochemical systems. (2) Nitrogen removal performance is quantitatively presented using: (a) nitrogen removal efficiency (%), which represents the percentage of influent nitrogen removed; and (b) nitrogen removal rate (g N m^−3^ d^−1^), which quantifies the volumetric nitrogen removal capacity. (3) CE represents coulombic efficiency (%), which reflects the ratio of electrons recovered as electrical current to the theoretical electrons available from the target substrate transformation. The calculation follows: CE (%) = (∫I dt/(n × F × ΔS)) × 100%, where *I* is the measured current (A), ∫I dt is the total charge transferred (C), *n* is the number of electrons transferred per mole of substrate, F is Faraday‘s constant (96,485 C mol^−1^), and ΔS is the amount of substrate transformed (mol). Some studies report CE values exceeding 100%, which typically indicate additional electron sources such as organic carbon, or an imbalance between the assumed and actual electron consumption. (4) Direct cross-study comparisons are complicated by variability in experimental conditions. Therefore, data in this table should be interpreted in the context of the original experimental conditions, and the reported values should be considered indicative of achievable ranges rather than directly comparable absolute performances across configurations. (5) Voltages refers to total applied voltage or the potential of a single electrode measured against a reference electrode, e.g., Standard Hydrogen Electrode (SHE) or Ag/AgCl.

## Data Availability

Data will be made available on request.

## Abbreviations

The following abbreviations are used in this manuscript:

BES	Bioelectrochemical system
EET	Extracellular electron transfer
Anammox	Anaerobic ammonium oxidation
TN	Total nitrogen
ESBR	Electrode-enhanced sequencing batch reactors
NH_4_^+^	Ammonium
Feammox	Ferric ammonium oxidation
SBR	Sequencing batch reactors
N_2_	Dinitrogen gas
N_2_H_4_	Hydrazine
NH_2_OH	Hydroxylamine
NH_4_^+^-N	Ammonia-nitrogen
HDB	Heterotrophic denitrifying bacteria
C/N	Carbon-to-nitrogen
H-ADB	Hydrogenotrophic-autotrophic denitrifying bacteria
MFC	Microbial fuel cell
H_2_	Hydrogen
DO	Dissolved oxygen
CW	Constructed wetland
CW-BES	Constructed wetland-bioelectrochemical system
CBSAD	Sulfur-based autotrophic denitrification
SMP	Soluble microbial products
PPy	Polypyrrole
EPS	Extracellular polymeric substances
Fe^2+^	Ferrous ions
SHE	Standard Hydrogen Electrode
Ag/AgCl	Ag/AgCl Electrode
